# Production of NiMn2O4 hollow spheres and CoFe_2_O_4_ bowl-like structures by using block copolymer stabilized polystyrene spheres as a hard template

**DOI:** 10.3906/kim-2106-18

**Published:** 2021-09-16

**Authors:** Gökhan KOÇAK, Vural BÜTÜN

**Affiliations:** 1 Department of Chemistry and Chemical Process Technologies, Vocational School of Higher Education, Adıyaman University, Adıyaman, Turkey; 2 Department of Chemistry, Faculty of Science and Letters, Eskişehir Osmangazi University, Eskişehir, Turkey

**Keywords:** Block copolymer, polystyrene latex, dispersion polymerization, hollow spheres, NiMn_2_O_4_, CoFe_2_O_4_

## Abstract

The aim of this study is to highlight the use of polystyrene (PS) latexes stabilized with block copolymers as a hard template in the production of metal oxide hollow spheres. PS latexes produced by dispersion polymerization by stabilizing with tertiary amine methacrylate-based diblock copolymer were used as a hard template in the preparation of nickel manganese oxide (NiMn_2_O_4_) hollow spheres and cobalt iron oxide (CoFe_2_O_4_) bowl-like structures. Thanks to the diblock copolymer stabilizer with tertiary amine functional groups on the PS surface, precursor salts of CoFe_2_O_4_ and NiMn_2_O_4_ were first homogeneously deposited on the surface of PS latexes with a controlled precipitation technique. Then, metal oxide hollow spheres and bowl-like structures were produced by calcination. XRD results showed that CoFe_2_O_4_ and NiMn_2_O_4_ structures were successfully obtained after calcination. The thermogravimetric analysis results showed that the CoFe_2_O_4_ and NiMn_2_O_4_ contents of the hybrid PS spheres were in the range of 26.0–28.6 wt%. SEM images showed that the inorganic-polymer spheres fused with each other after calcination to form larger magnetic CoFe_2_O_4_ bowl-like structures. SEM images also indicated successful production of highly rough NiMn_2_O_4_ hollow spheres with nanosheets on the surface.

## 1. Introduction

Nano- and microsized metal oxides, especially used in catalysis applications, are very popular structures in material science. Among them, inorganic hollow and bowl-like micro/nanostructures, a special class of materials, are good candidates for many applications due to their large surface area, low density and large amount of interior space compared to their solid counterparts, as well as other optical and catalytic properties [1–6]. In addition, various magnetic metal oxides such as α-/γ-Fe_2_O_3_, Fe_3_O_4_, Co_3_O_4_, CoFe_2_O_4_, NiMn_2_O_4_ and NiFe_2_O_4_ can be produced [7–10]. The magnetic nature of these materials provides them with significant advantages, such as being able to be directed in the magnetic field, as well as the reduction of repetitive use and physical losses, especially in catalysis and adsorption applications [7–9]. For practical applications, it is important to produce hollow spheres and bowl-like structures in the desired size, monodisperse size distribution, repeatable and cost-effective [1–5]. Using many different approaches, it is possible to produce uniform and repeatable hollow spheres and bowl-like structures [1–4]. Among these techniques, the use of hard templates (polymer, silica and carbon) is conceptually the simplest [1,2]. Polystyrene (PS), PS derivatives, poly(methyl methacrylate) (PMMA) and formaldehyde resins are often used as polymeric hard templates due to their easy and low cost [1,2].

Polymeric spheres have been designed using different strategies, with the necessity of having groups that enable interactions on the surface of the polymeric spheres produced, in order to accumulate inorganic compounds. One of them is the spheres produced by the emulsifier-free emulsion polymerization method, and the functionality in such structures is due to the anionic or cationic structure of the radical initiator used [11,12]. Other widely preferred method is to modify the surface of PS spheres using sulfuric acid [2,11,13]. Another polymeric hard screen approach is the production of spherical brushes by polymerization initiated from the polymer surface [14–17]. Many different techniques such as photodeposition, chemical vapor deposition, electrodeposition, controlled precipitation, hydrothermal deposition, electrostatic layer-by-layer (LbL) can be used for the coating of the surface of template materials with inorganic species [1–5]. After coating process, the hollow structures are obtained by dissolving the polymeric structure in a suitable solvent or by calcination [1–5]. With a pioneering approach proposed by us before, it is the use of block copolymer stabilizers that provide the basis for the interaction of inorganic species with PS spheres, allowing the accumulation of inorganic species on the PS surface. In our previous studies, we reported successful production of double layer nickel oxide and manganese oxide hollow spheres with a very rough surface with nanosheets on the surface [18], nickel oxide [19] and nickel iron oxide [20] hollow spheres.

In the present study, nickel manganese oxide (NiMn_2_O_4_) hollow spheres and cobalt iron oxide (CoFe_2_O_4_) bowl-like structures were produced by using block copolymer stabilized PS latexes as a hard template. The PS spheres with various size were produced by using poly[2-(diisopropylamino)ethyl methacrylate]-*block*-poly[2-(dimethylamino)ethyl methacrylate] (PDPA-*b*-PDMA), poly[2-(diethylamino)ethyl methacrylate]-*block*-poly[2-(dimethylamino)ethyl methacrylate] (PDEA-*b*-PDMA) and poly[2-(dimethylamino)ethyl methacrylate]-*block*-poly[2-*N*-mopholinoethyl methacrylate] (PDMA-*b*-PMEMA) diblock copolymers as stabilizer via dispersion polymerization. Thanks to the functionality provided by the tertiary amine methacrylate containing block copolymer fringes on the PS surface, it has been homogeneously coated (or deposited) with metal oxide precursor salts (metal hydroxides) in the presence of urea with the controlled precipitation technique. Finally, both polymeric compounds were removed and metal oxides were converted to CoFe_2_O_4_ and NiMn_2_O_4_ structures by calcination process. As stated above, PS spherical latexes stabilized with block copolymer, which can be produced in a simpler and more functional way than polymeric spheres to be used for this purpose, are candidates to be a new model in the production of different types of metal oxide hollow spheres.

## 2. Experimental section

### 2.1. Materials

2-(Diisopropylamino)ethyl methacrylate (DPA, SI-AL), 2-(dimethylamino)ethyl methacrylate (DMA, SI-AL), 2-(diethylamino)ethyl methacrylate (DEA, Aldrich) and 2-*N*-mofolinoethyl methacrylate (MEMA, Polysciences Inc.) monomers were first passed through the basic alumina column (SI-AL). Then, 2,2-diphenyl-1-picrylhydrazyl (DPPH) and granular calcium hydride were added and stored at −18 °C in a freezer. The monomers were distilled under vacuum before use. 1-Methoxy-1-trimethylsiloxy-2-methyl-1-propene (MTS), which is used as the initiator of the group transfer polymerization (GTP), was distilled under vacuum at room temperature. Tetra-n-butyl ammonium bibenzoate (TBABB) as a catalyst was synthesized in accordance with the literature [21]. THF was first dried with the addition of finely chopped solid sodium pieces by stirring 3 days at room temperature. It was then refluxed under dry nitrogen in the presence of solid potassium and used as a solvent in the polymerization reaction. PDPA-*b*-PDMA, PDEA-*b*-PDMA and PDMA-*b*-PMEMA diblock copolymers were synthesized by using group transfer polymerization technique as described before [22]. *n*-Pentane (Merck) was used to remove homopolymer contaminants from the diblock copolymers before proton NMR spectroscopy measurements.

Styrene (Merck) as a monomer, 2,2′-azodiisobutyronitrile (AIBN, Across) as a radical initiator, 1-propanol (SI-AL) and methanol (SI-AL) as a solvent and a diblock copolymer as stabilizer were used in the production of PS spheres via dispersion polymerization. Fe(NO_3_)_3_.9H_2_O (Panreac), Co(NO_3_)_2_.6H_2_O (Merck), MnCl_2_ (Merck), Ni(NO_3_)_2_.6H_2_O (Merck) were used for coating the surface of PS spheres. Urea (SI-AL) has been used in the production of spherical inorganic-polymer materials for the purpose of controlled precipitation.

### 2.2. Instrumentation

Molecular weight distributions (M_w_/M_n_) and number average molecular weights (M_n_) of the polymers were measured using gel permeation chromatography (GPC) having the following parameters: An Agilent Iso Pump 1200 Series and a refractive index detector, connected to PLgel Mixed-D and Mixed-E (5 and 3 μm, respectively, 300 mm × 7.5 mm, Polymer Laboratories, Amherst, MA) columns and eluted with HPLC-grade tetrahydrofuran that was stabilized with BHT (0.5 g L^−^^1^) and TEA (0.02%) at a flow rate of 1.0 mL min^−^^1^. PMMA standards (ex. Polymer Labs, M_n_: 1100–220000 g mol^−^^1^) were used for calibration. The comonomer ratio of PDPA-*b*-PDMA, PDEA-*b*-PDMA and PDMA-*b*-PMEMA diblock copolymers were determined from proton NMR spectra (deuteron solvents) by comparing related peaks of both blocks. The hydrodynamic diameters (R_h_) and polydispersity index values (PDI or *μ*_2_/G^2^) of PS spheres were determined by dynamic light scattering (DLS). DLS studies were performed using the ALV/CGS-3 compact goniometer system (Malvern, Inc, UK). This goniometer system is equipped with a 22 mW He-Ne laser at λ_0_ 632.8 nm, a photodiode detector operating with high quantum efficiency and an ALV/LSE-5003 multitau digital correlator electronic system. All measurements were made with 90° constant angle scattering of polymer dispersions. The data were evaluated by second order cumulative analysis. The solution temperature was kept constant at ±1 ºC sensitivity with a temperature controlled water bath. The wt% ratio of metal oxide or composites of metal oxide structures were determined with thermogravimetric analysis (TGA) device (Seiko SII Extar 6000 TG/DTA). Measurements were performed at different heating rates (1–10 ºC min^−1^) and under a flowing dry air atmosphere of 2 mL min^−1^. Protherm furnaces PAF 110/10 muffle furnace was used in the calcination process. The morphologies of PS latexes and metal oxide hollow spheres prepared were examined by light microscope (Leica DM750), and scanning electron microscope (SEM, Zeiss Evo LS10). Powder diffraction patterns of metal oxide hollow spheres were determined by X-ray diffraction (powder-XRD, PANalytical Empyrean) analysis using Cu Kα-radiation (λ¼ 1.54 Å) with 2θ angle in the range of 1–90 at room temperature. The powder diffraction patterns were examined in the HighScore Plus software and the peak determinations were made, and the phase content of the sample was illuminated with the reference phases found by searching from the ICDD PDF4+ library.

### 2.3. Production of PS microspheres by dispersion polymerization

**Figure 1:**
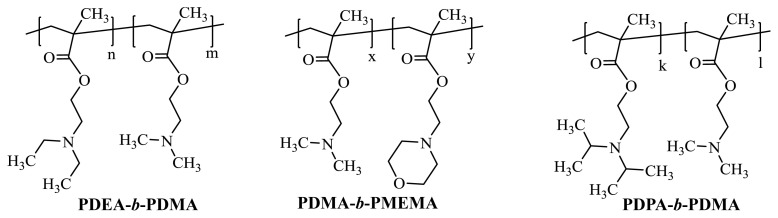
Chemical structures of the diblock copolymer stabilizers.

In this study, PDPA-*b*-PDMA, PDEA-*b*-PDMA [22] and PDMA-*b*-PMEMA [23] diblock copolymers, which we previously produced and characterized by GTP method, were used as stabilizers (Figure 1). The detail of the production of PDPA-*b*-PDMA diblock copolymer with GTP, which is used as a stabilizer in the synthesis of PS spheres, is given in the supporting information.

**Figure 2:**
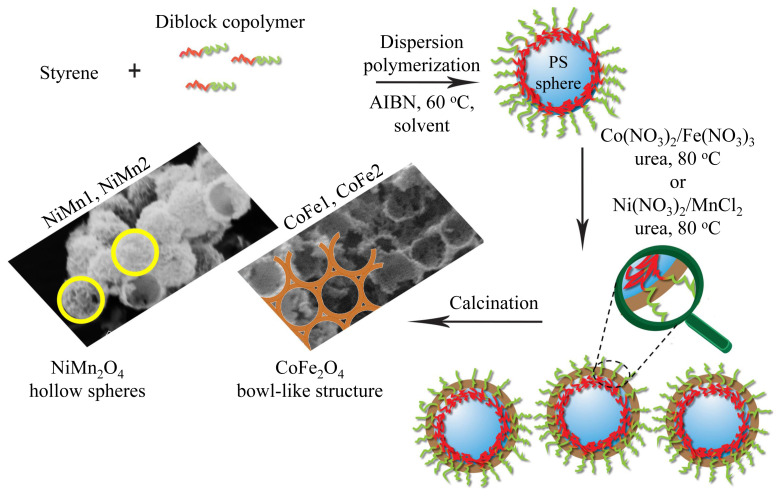
Schematic representation of the production of NiMn_2_O_4_ hollow spheres and CoFe_2_O_4_ bowl-like structures using PS spheres stabilized with block copolymer.

Synthesis of PS microspheres (latexes) was performed using PDPA_0.17_-*b*-PDMA_0.83_ diblock copolymer (1.0 g, 25500 g mol^−1^, M_w_/M_n_: 1.08) stabilizer, AIBN initiator (50 mg), styrene monomer (5.0 mL), 1-butanol or H_2_O/methanol (50.0 mL) under nitrogen atmosphere in oil bath at 60 °C at 1000 rpm stirring speed (Figure 2). The reaction was continued overnight. The solution was centrifuged twice at 10000 rpm for 10 min to precipitate PS microspheres. PS spheres were dried overnight in vacuum. Experimental conditions of other produced PS spheres are given in Table 1. The spheres were characterized by DLS after centrifugation.

### 2.4. Preparation of metal oxide structures

Schematic representation of the production of NiMn_2_O_4_ hollow spheres and CoFe_2_O_4_ bowl-like structures using PS spheres stabilized with block copolymer are given in Figure 2. The deposition of metal hydroxides on the surface using the controlled precipitation technique in the presence of urea and then the production of both CoFe_2_O_4_ [9, 24–27] and NiMn_2_O_4_ [28, 29] structures by calcination process have been studied previously. The production of metal oxides was carried out very similar to these previous studies.

**Figure 3:**
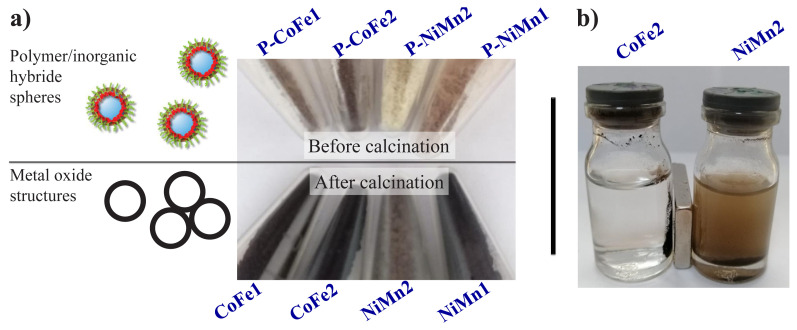
Digital pictures of PS-inorganic hybrid spheres (before calcination) and metal oxide structures (after calcination) (a) and the behavior of metal oxide species in a magnetic field (b).

First, PS spheres (0.2 g) were dispersed in 180.0 mL of water (pH 7.0). The amounts of Fe(NO_3_)_3_ and Co(NO_3_)_2_ solutions given in Table 2 were then added and stirred for 24 h. Finally, urea was dissolved in water (20.0 mL) and added to the reaction medium and stirred at 80 °C for 24 h. Similar process was carried out using Ni(NO_3_)_2_ and MnCl_2_ solutions, and all details are given in Table 2. The products were centrifuged three times at 5000 rpm and washed three times with distilled water. It was seen that the centrifuged solution part was completely clear, that is, all metal oxide precursor salt deposited on the PS surface in a controlled precipitation. It was observed that the PS spheres coated with both metal oxide precursor salts turned from white to brown tones over time (see Figure 3). The resulting material was dried in an oven at 100 °C overnight. The productions of all metal oxide structures are given in Table 2.

Finally, PS-inorganic hybrid spheres containing Co/Fe and Ni/Mn were calcinated at 700 ºC for 1 h and at 600 ºC for 2 h in air atmosphere, respectively. In these calcination processes in the furnace, both types of hybrid spheres were heated up to 300 ºC and kept at this temperature for 1 h, removing most of the polymeric structure. Then it was heated from 300 ºC to calcination temperatures (600 or 700 ºC) with a heating rate of 1 ºC/min and the calcination process was terminated by keeping at this temperature. The difference in the colors of the hybrid spheres before the calcination and the metal oxide structures formed after the calcination is given in Figure 3.

**Figure 4:**
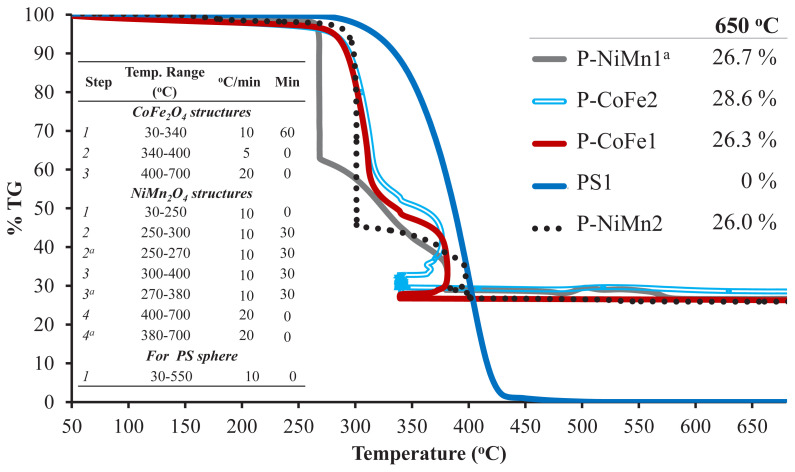
TGA chromatograms of PS sphere (PS1) andmetal oxide structures.

The wt% ratios of metal oxides in PS-inorganic hybrid spheres were determined with by thermogravimetric analysis (TGA). TGA measurements were carried out in a dry air atmosphere (2 mL min^−1^) and the heating program applied in the measurements is given in Figure 4. It was aimed to remove polymeric parts by keeping at approximately 300 ºC for 60 min in TGA measurements of hybrid spheres as in the calcination process performed in the furnace. Since deviation was observed in TGA chromatograms due to intense combustion, it was planned to be kept at these temperatures for 60 min and to completely burn the polymeric part in a controlled manner. However, unexpected fluctuations were observed in TGA chromatograms, which we thought to be due to the polymer not being completely removed. The contents and morphology of the produced metal oxide structures were determined by XRD, light microscope and SEM.

## 3. Results and discussion

### 3.1. Production of PS microspheres

The tertiary amine methacrylate based PDPA-*b-*PDMA, PDEA-*b*-PDMA and PDMA-*b*-PMEMA diblock copolymers each served as a good dispersing agent in the production of PS spheres and enabled the production of monodisperse PS spheres (Table 1). In PS latex stabilization using this block copolymer, the less soluble PDPA, PDEA or PMEMA block are adsorbed on the latex surface, while the more soluble PDMA blocks are responsible for the stabilization of latexes. The polymer chains belonging to the PDMA block are located in the form of fringes in the shell of PS spheres, just like spherical brushed polymers.

Polymeric spheres stabilized with block copolymers have been preferred by polymer scientists to produce more monodisperse or environmentally sensitive spherical particles [30–34]. The resulting spherical particles exhibit changes in swelling-shrinkage behavior or surface properties with external stimuli such as temperature and pH [30–34]. In other words, interactions are established between block copolymers and polymeric spheres used as stabilizers in emulsion and dispersion polymerization techniques. These interactions are more stronger in emulsion polymerization, but some of these block copolymers remain on the surfaces of latex after their synthesis via dispersion polymerization as well [30,34,35].

As a result of DLS studies, hydrodynamic radius (R_h_) values and polydispersity index values (*μ*_2_/G^2^) of PS spheres stabilized with different block copolymers are given in Table 1. According to these results, it can be said that PS spheres are produced as monodisperse with a diameter of 1–2 μm. There are many studies showing that PS spheres can be produced in planned diameters by changing many factors such as heterogeneous polymerization technique, stabilizer type, stabilizer amount, mixing speed and solvent type [30–36]. When the previous studies are examined, if the amount of stabilizer increases or the mixing speed increases, the diameter becomes smaller. Comonomer ratios in the block copolymer have a significant effect on the diameter [23]. The type of solvent and solvent mixtures are also very effective on diameter change [36]. Since the comonomer ratios and molecular weights of the stabilizers used in the production of each PS sphere are different, it will be very difficult to compare with each other. However, it is well known that the ratios, lengths, hydrophilic/hydrophobic nature of the blocks in the stabilizer structure are decisive in ensuring that the PS diameters are at the desired size.

### 3.2. Preparation of metal oxide structures

In the previous section, it was mentioned that block copolymers used as stabilizers in dispersion (or emulsion) polymerization adhere to the surface [30,34,35]. The polymer fringes with this DMA unit provided a suitable environment for the absorption of metal ions. However, in this way, the metal oxide precursor salt can be deposited or adsorbed homogeneously on the structure used as a hard template. Incidentally, it is also known that PS spheres are frequently used as a hard template in the coating of polymeric cores with an inorganic layer [1,2]. PS spheres are preferred because the phenyl ring is modifiable, easy to prepare, and is a low cost polymer that is easily available commercially. In addition, poly(methyl methacrylate) and formaldehyde resin are other common polymers used for this purpose [1,2].

Together with our previous studies [18–20], it will be very useful for the reader to compare diblock copolymer stabilized PS spheres which is used as a hard template with other polymeric rigid templates in terms of functionality and production technique in this pioneering work. Inorganic species must have a surface charge (or functionality) in order to adsorb to the surface of PS spheres. This can only be achieved with stabilizing agent [30–34], anionic radical (ammonium persulfate and potassium persulfate) and cationic radical [2,2′-azobis(2-methylpropionamidine) dihydrochloride] initiators used in the emulsifier-free polymerization method [11,12], various modifications made on the phenyl ring in PS spheres [2,11,13], and spherical polymeric brush polymers, which are surface initiated polymerization products [14–17]. In addition, the fact that PS spheres have a charged surface is important in that it allows the coating of PS spheres with inorganic species with the layer-by-layer coating (LbL) technique [37]. Polymeric spheres stabilized with block copolymers contain polymer fringes around them, just like brush polymers, can be produced quite simply compared to brush polymers which are surface-initiated polymerization products that require special monomers or various modifications [14–17]. It is worth to mention that these polymeric spherical brushes are frequently used in the production of inorganic nanoparticles (NP) rather than the production of hollow spheres [14–17]. On the other hand, it is quite common to use anionic PS spheres formed by the sulfonation of the polystyrene surface with the H_2_SO_4_ treatment, but it can still be said that the PS spheres stabilized with the block copolymers used in this study are more functional [2,11,13]. It can be assumed that block copolymers on the surface of PS spheres provide adsorption of inorganic species to the surface and provide a completely homogeneous coating by preventing separation from the surface by forming a steric barrier during nucleation-growth [14–17].

In this study, it was thought that the mechanism of action of the diblock copolymer used to stabilize PS spheres was to establish interaction with metal ions, increase the concentration of metal ions on the PS surface with adsorption, form nuclei on the surface of the inorganic species in the basic medium, and the growth of the crystals of metal oxide precursor salts on the PS surface thanks to the polymer fringes. The fact that tertiary amine methacrylate based polymers and many other polymers have already been discussed in many studies as metal ion adsorbents [38,39]. However, it should be kept in mind that many different types of block copolymers can be designed and used for this purpose [38,39]. It is also important that the polymer to be selected for this purpose has to have high metal ion adsorption capacity, low cost and easy availability.

Using the controlled precipitation technique as in this study, many metal oxide hollow spheres and bowl-like structures such as CuO, ZnO, SnO_2_, CeO_2_, MgO, *α*-Fe_2_O_3_, Cr_2_O_3_, In_2_O_3_, Co_3_O_4_, NiO, CoFe_2_O_4,_ NiFe_2_O_4_ and other [6,40] can be produced for different applications. To summarize briefly, urea added to the reaction mixture slowly decomposes to NH_3_ at 80 ºC, that is, the hydroxide ion concentration in the mixture increases and metal hydroxides begin to precipitate in the PDMA fringes on the PS surface. In other words, Fe(OH)_3_/Co(OH)_2_ and Mn(OH)_2_/Ni(OH)_2_ crystals are grown in a controlled manner on the PS surface. It is then converted to CoFe_2_O_4_ and NiMn_2_O_4_ by thermal decomposition (calcination) and polymeric compounds are removed at this time [9,24–28].

According to the results of thermogravimetric analysis (TGA), it was observed that 26.0–28.6 wt% residue remains at 650 ºC (Table 2). Considering that PS spheres did not leave any residue at the same temperature, almost all of the structures formed after the calcination of polymer-inorganic hybrid structures belonged to CoFe_2_O_4_ and NiMn_2_O_4_ residues (Figure 4). Of course, by adding higher proportions of precursor metal salts, these residue amounts can be further increased, and this change causing an increase in the shell thickness contributes to the hollow spheres remaining unbreakable.

**Figure 5:**
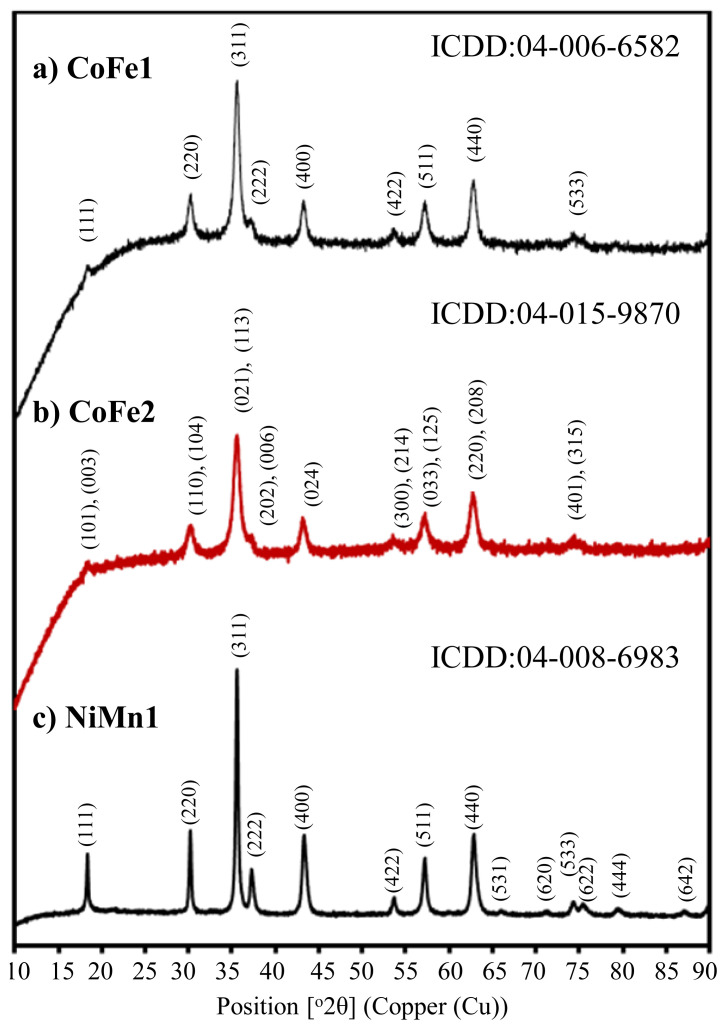
X-ray diffraction pattern of the prepared CoFe_2_O_4_ and NiMn_2_O_4_ structures.

Determination of the crystal phase identification of the synthesized CoFe_2_O_4_ and NiMn_2_O_4_ structures was done with XRD analysis. After 1 h of calcination at 700 ºC, it was determined that CoFe1 and CoFe2 were cobalt iron oxide (CoFe_2_O_4_) with cubic and rhombohedral crystal structure, respectively. The XRD pattern of CoFe1 shows major diffraction peaks positioned at 2θ of 18.34º, 30.27º, 35.60º, 37.16º, 43.27º, 53.66º, 57.23º, 62.77º and 74.34º correspond to the planes (111), (220), (311), (222), (400), (422), (511), (440) and (533), respectively, as seen in Figure 5a. This XRD pattern is well matched with the standard ICDD: 04-006-6582. The XRD pattern reveals that the synthesized metal oxide hollow spheres are in the CoFe_2_O_4_ phase with a cubic crystal structure belonging to the space group Fd-3m. The obtained XRD pattern CoFe2 shows major diffraction peaks positioned at 2θ of 18.35º, 30.21º, 35.58º, 37.07º, 43.12º, 53.50º, 57.16º, 62.72º and 74.17º correspond to the planes (101 and 003), (110 and 104), (021 and 113), (202 and 006), (024), (300 and 214), (033 and 125), (220 and 208) and (401 and 315), respectively, as shown in Figure 5b. This XRD pattern is well matched with the standard ICDD: 04-015-9870. The obtained XRD pattern reveals that the synthesized metal oxide hollow spheres are in the CoFe_2_O_4_ phase with a rhombohedral crystal structure belonging to the space group R-3m. After 2 h of calcination at 600 ºC, it was determined that nickel manganese oxide (NiMn_2_O_4_) was formed in cubic (ICDD: 04-008-6983) crystal structure. The obtained XRD pattern of NiMn1 shows major diffraction peaks positioned at 2θ of 18.35º, 30.19º, 35.58º, 37.24º, 43.3º, 53.65º, 57.18º, 62.84º, 66.01º, 71.16º, 74.25º, 75.37º, 79.44º, and 87.10º correspond to the planes (111), (220), (311), (222), (400), (422), (511), (440), (531), (620), (533), (622), (444), and (642) respectively, (see Figure 5c). This XRD pattern is well matched with the standard ICDD: 04-008-6983. The obtained XRD pattern reveals that the synthesized metal oxide hollow spheres are in the MnNi_2_O_4_ phase with a cubic crystal structure belonging to the space group Fd-3m. As seen in Figure 5, there is no other peaks related to cobalt oxide, nickel oxide, manganese oxide, iron oxide or other phases which indicate that we have pure CoFe_2_O_4_ [26] and NiMn_2_O_4_ [41].

**Figure 6:**
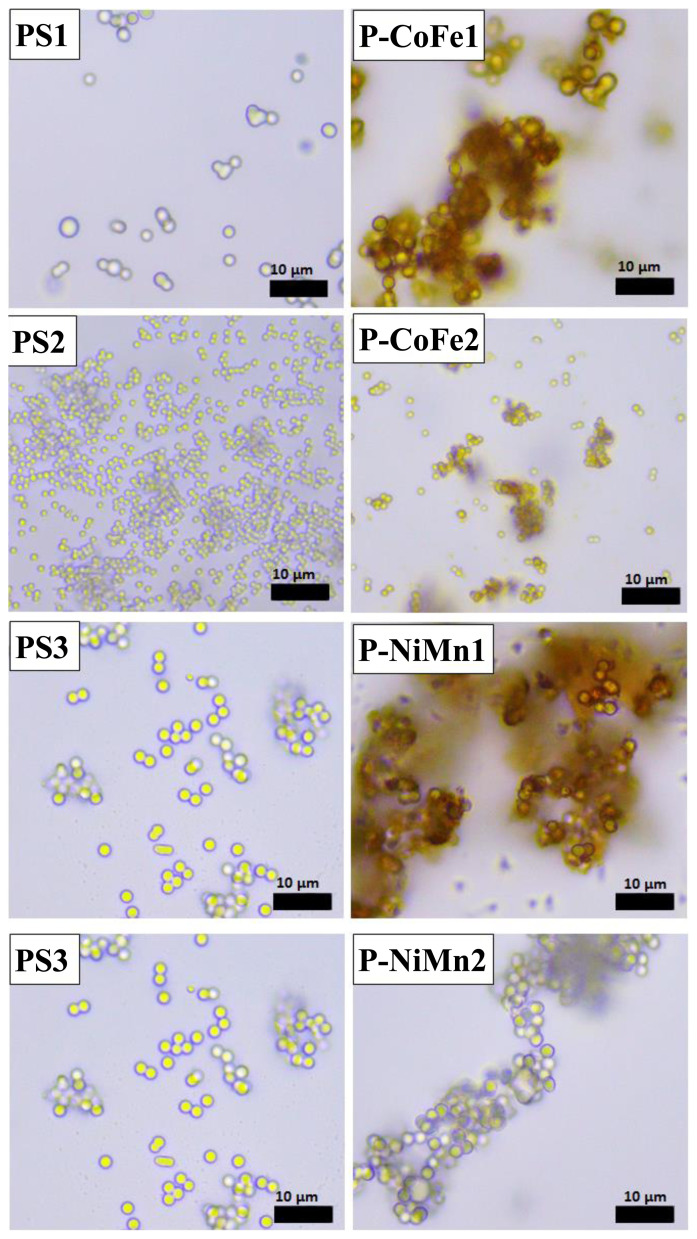
Light microscope images of PS spheres and inorganic-PS hybrid spheres (before calcination).

**Figure 7:**
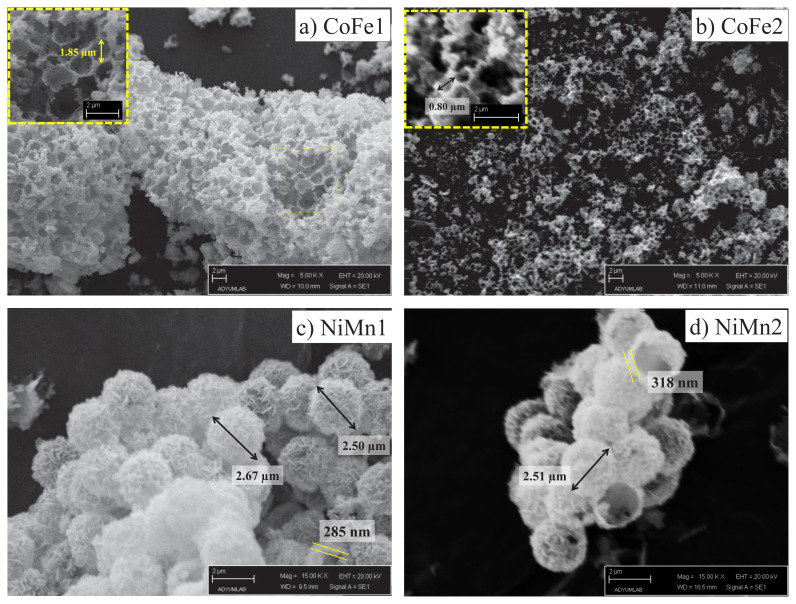
SEM images of CoFe_2_O_4_ (CoFe1 and CoFe2) and NiMn_2_O_4_ (NiMn1 and NiMn2) structures produced after calcination.

It can be easily understood by comparing the light microscope images of PS spheres and inorganic-PS hybrid spheres that the surfaces of all PS spheres are successfully homogeneously coated with Fe(OH)_3_/Co(OH)_2_ and Ni(OH)_2/_Mn(OH)_2_ (Figure 6). Again, these images showed that no other precipitate structures were formed except for the PS surface (Figure 6). By taking SEM images of metal oxide structures, both their homogeneity and more detailed morphological structures were revealed. SEM images of the metal oxide structures taken after calcination also indicated that the PS spheres were coated homogeneously (Figure 7). From the SEM images of CoFe1 and CoFe2, it was seen that the inorganic-PS hybrid spheres fused with each other after calcination process to form larger structures (Figures 7a and 7b). Magnetic CoFe_2_O_4_ hollow bowl-like structures were obtained in the outer layer of this structure, and hollow spheres were obtained in the inner layers. The formation of these bowl-like structures resulted from the mechanical abrasion of the hollow spheres in the outer layer. The diameters of the bowl-like structures formed on the surface were, as expected, approximately 1.85 μm for CoFe1 and approximately 0.80 μm for CoFe2, in relation to the diameters of the PS spheres used. In another study where polymeric spheres were used as a rigid template and similar metal oxide type was also similar, structures with similar morphological properties were obtained, which revealed the effect of temperature [42]. Although the first structure planned to be produced is metal oxide hollow spheres, the obtained bowl-like structures can be related to the nature of the metal oxide as well as the calcination temperature and time. It is possible to produce metal oxide hollow spheres by experimenting with different calcination temperature and time [42,43]. SEM images of NiMn1 and NiMn2 samples showed that NiMn_2_O_4_ hollow spheres with a diameter of approximately 2.50 μm were successfully produced (Figures 7c and 7d). The reason why both have similar diameters is, of course, because they are produced using the same PS template. SEM images showed that the shell thicknesses of NiMn1 and NiMn2 hollow spheres were approximately 285 nm and 318 nm, respectively (Figure 7). The difference between NiMn1 and NiMn2 is the amount of urea and a change in the color of the resulting inorganic-PS hybrid sphere was observed (Figure 3). There were nanosheets on the surface of both NiMn_2_O_4_ hollow spheres. It has been emphasized in previous studies that the presence of nanosheets has an effect on increasing the surface area of the structure [44].

The cobalt iron oxide (CoFe_2_O_4_) is an important type of metal oxide that has applications in various fields such as sensor [45], photocatalysts [8,9], electrocatalyst [46], cancer therapy [47], batteries [25,27], magnetic optical behavior [48] and supercapacitors [49]. It is n-type semiconductor, highly stable, small optical band gaps (approximately 2.6 eV) making them active under visible light treatment [50]. The nickel manganese oxide (NiMn_2_O_4_) has been widely studied and applied in many fields such as sensor [51], negative temperature coefficient thermistors [52], photocatalysts [53], electrocatalyst [54], supercapacitors [28, 51], and batteries [55] owing to its various advantages, such as low cost, resource abundance, good stability, environmental friendliness, convenience in use and excellent electrochemical performance [56]. The effectiveness of the materials also depends on their morphology, size and composition of the materials. In this respect, it is undoubted that reproducible and uniform metal oxide structures with varying diameters depending on the choice of polymeric sphere used as template can be used in many similar applications with the positive effect of high surface area.

## 4. Conclusion

The PS spheres produced in different diameters by dispersion polymerization using different diblock copolymers as stabilizers were used in the production of NiMn_2_O_4_ hollow spheres and CoFe_2_O_4_ bowl-like structures. The dimensions of these structures formed according to the diameters of the spherical spheres also changed as expected.

This study reports successful usage of spherical PS latexes stabilized with tertiary amine methacrylate based diblock copolymer as a template, which offers a new approach in terms of the use of hard templates. The surfaces of PS spheres are surrounded by a hydrated PDMA block of steric stabilizer, tertiary amine methacrylate based diblock copolymers. These stabilizers give them the ability to adsorb inorganic species at a higher capacity and stabilize the formed seeds on the surface, allowing the homogeneous metal oxide precursor salt to accumulate on the surface. Moreover, it should be noted that these PS spheres can be produced with a wide variety of block copolymers. Such hard templates have important advantages such as having more functional groups than surface modified PS spheres and being prepared with a simpler technique compared to spherical PS brushes. It is quite possible that the spheres produced in this study and other inorganic hollow spheres we continue to produce will be used in various catalysis studies in the future.

## Supplementary material

### Synthesis of PDPA-*b*-PDMA diblock copolymer with GTP

The synthesis of PDPA-*b*-PDMA diblock copolymer has been performed as follows using group transfer polymerization [1]: First, a 250 mL three-necked flask taken from the oven at 130 °C was placed on the vacuum line and heated with a heat gun and vacuumed in high vacuum. Solid tetra-*n*-butyl ammonium bibenzoate (TBABB) catalyst (approximately 100 mg) was added into the three-necked flask in the presence of dry nitrogen and vacuumed again. A total of 160 mL of tetrahydrofuran (THF) as solvent and 0.55 mL of 1-methoxy-1-trimethylsiloxy-2-methyl-1-propene (MTS) as initiator, respectively, were transferred into the three-necked flask via cannula. After the initiator was activated by stirring the solution for 15 min, DPA monomer (10 mL), as first monomer, was added to the reaction medium to obtain the first block. In the meantime, the temperature change of the reaction medium was observed with a contact thermocouple attached to the surface of the balloon, and it was determined that the temperature increased by 4 °C with the exothermic polymerization. The polymer solution was stirred for 40 min at room temperature. At the end of 40 min, 1 mL of sample was taken from the medium for GPC and proton NMR analysis and was terminated with 0.1 mL of methanol. The second monomer, DMA (23 mL), was added to the reaction medium under nitrogen atmosphere via a cannula, just as in the first monomer addition. A second exotherm was observed (10 °C). The reaction was stirred at room temperature for about 1 h and at the end of this period, polymerization was terminated by adding 1 mL of methanol to the reaction medium.

GPC and proton NMR analyzes were performed by taking 1 mL sample. The solid polymer obtained by evaporating the polymer solution in a rotary evaporator was dried in the freeze dryer. Homopolymer residues were observed as a result of GPC. The polymer was dissolved in THF and precipitated in *n*-pentane to get rid of these residues. As can be seen from the GPC chromatograms of the homopolymer and diblock copolymer, polymers with very narrow molecular weight distribution (*M_w_/M_n_*) have been obtained and there is no homopolymer residue in the diblock copolymer (Figure S1). GPC result indicated the number average molecular weight (*M_n_*) and molecular weight distribution value of the diblock copolymer to be 25500 g mol^−1^ and 1.08, respectively.

End group analysis was performed to determine the composition (in mol%) and polymerization degree (DP) of the PDPA-*b*-PDMA diblock copolymer. In the synthesis, first the PDPA block was produced, then the second monomer, DMA, was added and the ^1^H NMR spectrum of the PDPA homopolymer was also taken just before addition of DMA monomer as given in Figure S2.

In order to determine comonomer ratios or mol percentages, the integral area of the isopropyl group C-H protons (**c** signal) of the PDPA at 2.94 ppm (see Figure S3) was compared with the integral area of the **a+g** signals of –C(=O)OCH_2_-protons belonging to both PDPA and PDMA blocks in the range of 3.64–4.21 ppm (see Figure S3). The DPA and DMA contents of diblock copolymer were determined to be 16.7 mol% and 83.3 mol%, respectively. Thus the block copolymer was expressed as PDPA_0.17_-*b*-PDMA_0.83_. This polymer was soluble in water at room temperature. It is soluble molecularly in acidic solution but it forms micelles in neutral water or in basic conditions by PDPA block forming the micellar core.^1^

Figure S1 GPC chromatograms of PDP Ahomopolymer (a) and PDPA-*b*-PDMA diblock copolymer (b).

Figure S2 ^1^H NMR spectrum of PDPA homopolymer in CDCl_3_.

Figure S3 ^1^H NMR spectrum of PDPA-*b*-PDMA diblock copolymer in CDCl_3_.

References1

ButunV
ArmesSP
BillinghamNC

Synthesis and aqueous solution properties of near-monodisperse tertiary amine methacrylate homopolymers and diblock copolymers
Polymer
2001
42
14
5993
6008
10.1016/s0032-3861(01)00066-0


## Figures and Tables

**Table 1:** Experimental conditions in the synthesis of the PS latexes with various size and DLS measurements (styrene 5.0 mL, 1000 rpm and at 60 ºC).

Code	Stabilizer type	Stabilizer amount	AIBN	Media (50 mL)	Diameter (nm)	*μ*_2_/G^2^
PS1	aPDPA_0.17_-*b*-PDMA_0.83_ bM_n_: 25500 gmol^−^^1^, PDI: 1.08	1.0 g	60 mg	H_2_O/MeOH (1/9)	2150	0.02
PS2	aPDMA_0.86_-*b*-PMEMA_0.14_ bM_n_: 45600 g mol^−^^1^, PDI: 1.13	0.6 g	45 mg	1-butanol	1050	0.08
PS3	aPDEA_0.30_-*b*-PDMA_0.70_ bM_n_: 14900 g mol^−^^1^, PDI: 1.06	0.6 g	45 mg	H_2_O/MeOH (1/12)	1400	0.06

a Mole% content determined by proton NMR spectroscopy.

b GPC results (THF eluent, PMMA standards).

**Table 2:** Experimental conditions in the synthesis of the inorganic-PS hybrid spheres (in 200 mL water, at 80 ºC).

Code	PS spheres (0.2 g)	Urea	Fe(NO_3_)_3_ (0.20 M)	Co(NO_3_)_2_ (0.20 M)	Residue at 650 ºC (wt%)
P-CoFe1	PS1	2.0 g	4.0 mL	2.0mL	26.3
P-CoFe2	PS2	2.0 g	4.0 mL	2.0mL	28.6
**Code**	**PS spheres (0.2 g)**	**Urea**	**Ni(NO** **_3_** **)** **_3_** ** (0.20 M)**	**MnCl** **_2_** ** (0.20 M)**	**Residue at 650 ºC (wt%)**
P-NiMn1	PS3	3.0 g	2.0 mL	4.0mL	26.7
P-NiMn2	PS3	4.0 g	2.0 mL	4.0mL	26.0
