# Supplementary figures and images for: Production of NiMn2O4 hollow spheres and CoFe2O4 bowl-like structures by using block copolymer stabilized polystyrene spheres as a hard template
Source: Turk J Chem. 2021 Sep 16;46(1):1–13. doi: 10.3906/kim-2106-18 (PMC10734705; doi:10.3906/kim-2106-18)

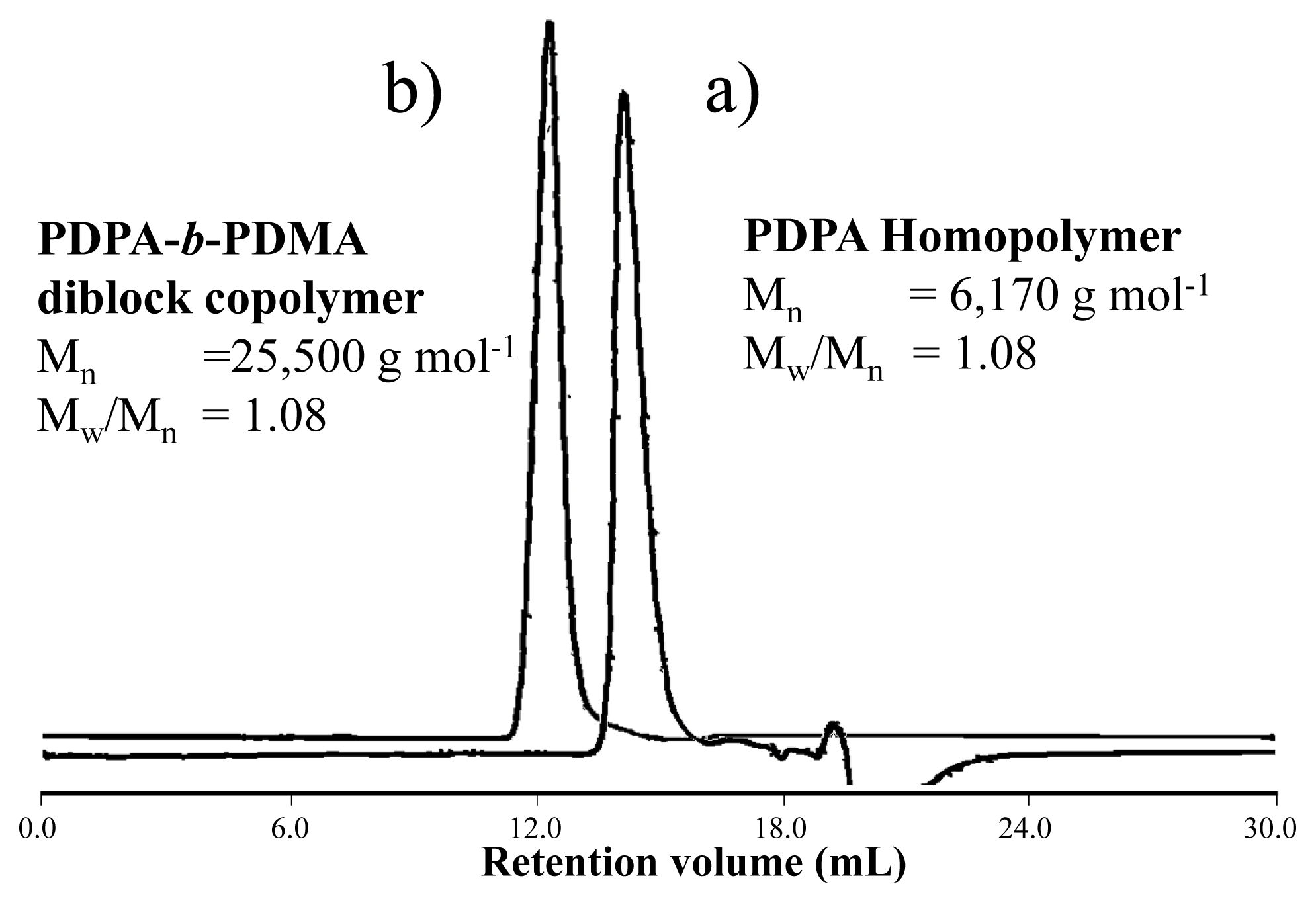

Supplement: Figure S1 — GPC chromatograms of PDP Ahomopolymer (a) and PDPA-b-PDMA diblock copolymer (b). [file turkjchem-46-1-1s1.tif]

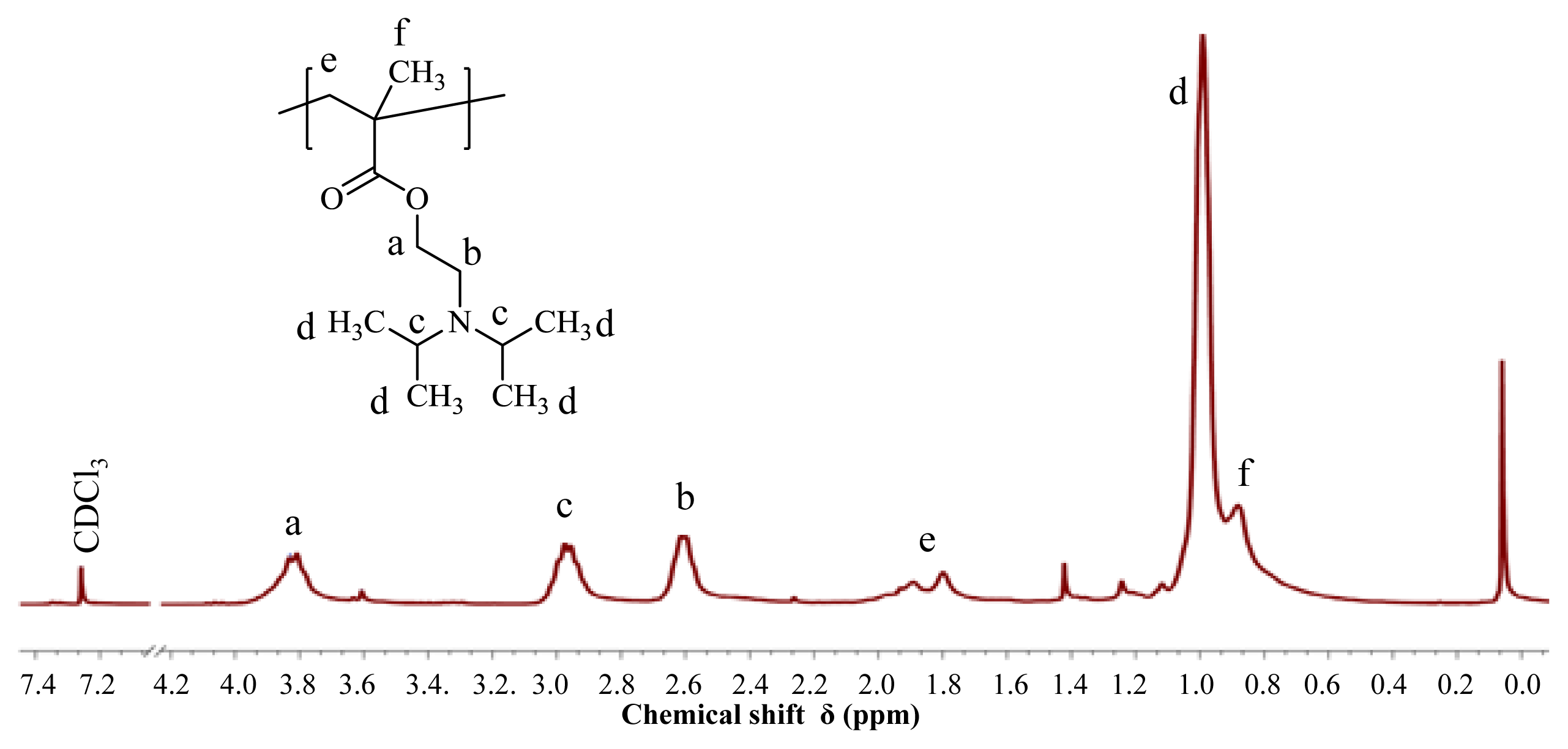

Supplement: Figure S2 — 1H NMR spectrum of PDPA homopolymer in CDCl3. [file turkjchem-46-1-1s2.tif]

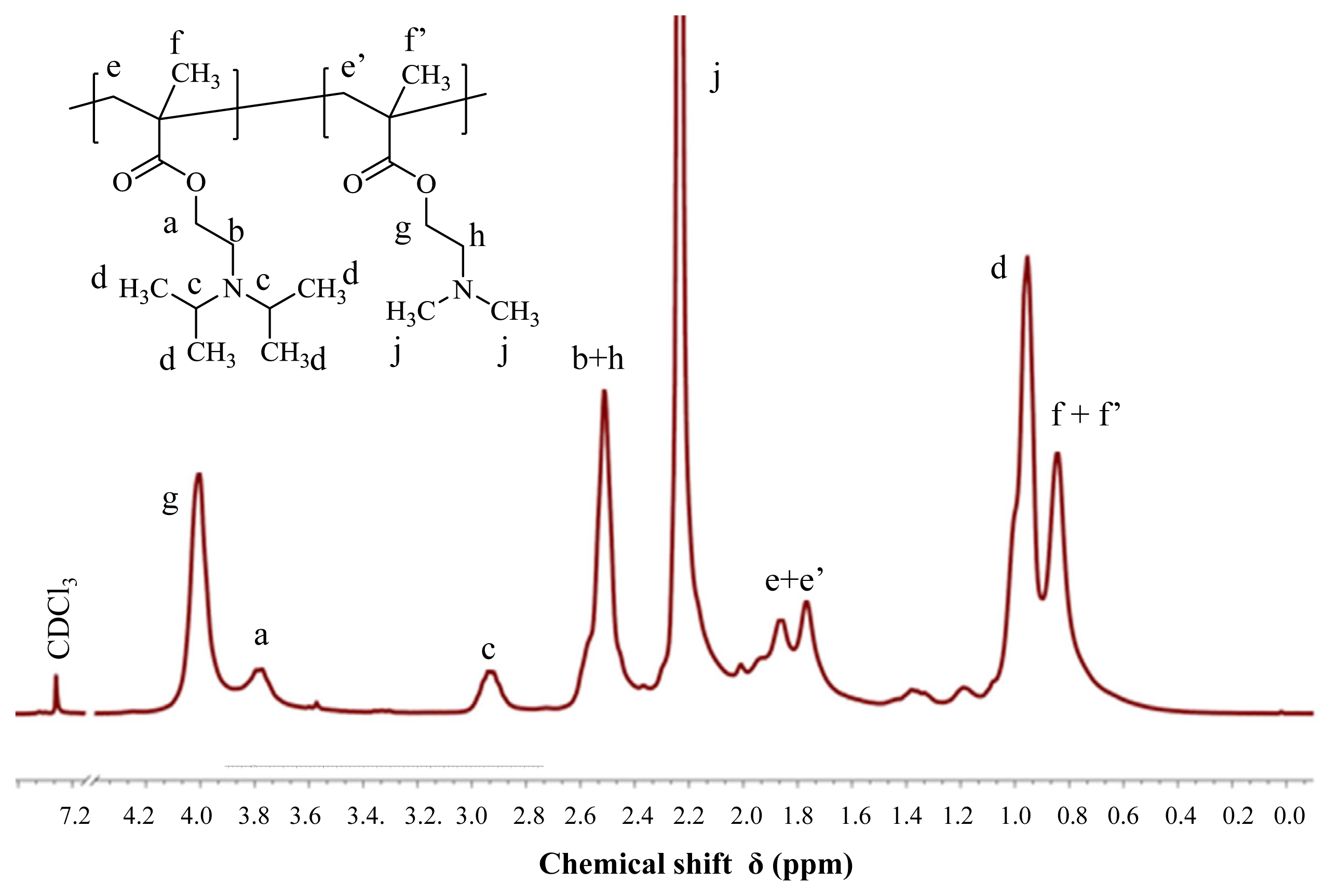

Supplement: Figure S3 — 1H NMR spectrum of PDPA-b-PDMA diblock copolymer in CDCl3. [file turkjchem-46-1-1s3.tif]
